# Nonadherence to the WHO 2016 Antenatal Care Model and Associated Factors Among Delivering and Postnatal Women in Southern Ethiopia: A Cross‐Sectional Study

**DOI:** 10.1155/jp/8584385

**Published:** 2026-07-24

**Authors:** Kassa Genetu, Genet Mekuye, Anteneh Gashaw, Addisu Getnet, Zerihun Figa

**Affiliations:** ^1^ Department of Midwifery, College of Medicine and Health Sciences, Dilla University, Dilla, Ethiopia, du.edu.et

**Keywords:** antenatal care, associated factor, nonadherence, pregnant women, southern Ethiopia

## Abstract

**Background:**

To reduce maternal and fetal mortality and morbidity, the World Health Organization (WHO) recommended eight or more antenatal care (ANC) contacts for pregnant mothers, known as the WHO 2016 ANC model. However, the effectiveness of the model depends on the adherence of pregnant mothers. Therefore, this study is aimed at assessing nonadherence to the 2016 ANC model and the associated factors among delivering and postnatal mothers in southern Ethiopia.

**Method:**

An institution‐based cross‐sectional study was conducted from August 25, 2024, to October 20, 2024, at Dilla University Teaching Hospital, Dilla town, southern Ethiopia. A systematic random sampling technique was used to select the study participants. A pretested questionnaire was used to collect data on KoboCollect, and data analysis was conducted using the Statistical Package for the Social Sciences (SPSS) Version 25. A descriptive analysis was conducted and presented in tables, graphs, charts, and bars. Bivariate and multivariate analyses were performed to identify associations between nonadherence to the 2016 ANC model and other factors. Multivariable analysis at the level of 95% confidence intervals of statistical significance was declared at a *p* value < 0.05.

**Result:**

In this study, 308 women participated, making a response rate of 95%. The mean age of study participants was 26.16 ± 5.51. Almost half (49.7%) of participants were housewives, 94 (30.5%) of the women attended secondary school, and 160 (51.9%) resided in urban settings. The study revealed that 45.5% (95% CI: 42.12, 56.3) of respondents were nonadherent to the WHO 2016 ANC model. Women who reside in rural settings (AOR: 2.4; 95% CL: 1.14–3.42), having no formal education (AOR: 2.4; 95% CI: 1.009–4.08), women′s husbands being the principal income generator of the household (AOR: 2.01; 95% CI: 1.009–4.08), and women′s husbands being head of the household (AOR: 11.53; 95% CI: 1.024–38.6) were significantly associated with nonadherence to the WHO 2016 model.

**Conclusion:**

We found that nearly half of laboring and postnatal women were nonadherent to the newly recommended WHO 2016 eight‐contact ANC model. Therefore, efforts should focus on women′s economic empowerment and education, with particular emphasis on raising awareness about the importance of attending frequent ANC visits. Future researchers should consider conducting qualitative studies to better explore the reasons behind nonadherence to ANC.

## 1. Introduction

Antenatal care (ANC) refers to the care provided by qualified healthcare professionals to pregnant women and adolescent girls to ensure optimal health for both the mother and baby during pregnancy [1]. Nonadherence to ANC occurs when pregnant individuals fail to attend scheduled appointments or follow recommended care protocols during their pregnancy. ANC encompasses a wide range of services offered by healthcare providers to pregnant women, aiming to monitor the well‐being of both the mother and fetus, detect and address pregnancy complications, address the mother′s concerns, prepare her for childbirth, and promote healthy behaviors [2, 3]. Despite its crucial role in improving maternal and newborn health outcomes, nearly 50% of women in low‐ and middle‐income countries (LMICs) do not receive adequate ANC [4, 5].

In 2020, approximately 287,000 women worldwide died from maternal causes, which averages to 800 maternal deaths each day, or one every 2 min. Sub‐Saharan Africa (SSA) had an exceptionally high maternal mortality ratio (MMR), responsible for about 70% of global maternal deaths [6]. Ethiopia, a country in SSA, also experiences high maternal mortality rates. The 2016 Ethiopian Demographic and Health Survey (EDHS) reported a MMR of 412 per 100,000 live births. Additionally, Ethiopia′s neonatal mortality rate increased, rising from 29 to 30 deaths per 1000 live births [7]. Local research conducted in Ethiopia in 2020 further supports this increment, showing a pooled prevalence of neonatal mortality at 6.78%, equivalent to approximately 67 deaths per 1000 live births [8].

The Sustainable Development Goal 3 (SDG3) seeks to reduce maternal mortality to fewer than 70 per 100,000 live births and neonatal mortality to below 12 per 1000 live births by 2030, a target that can be supported by ensuring access to adequate ANC services [8]. However, many women may be unaware of the new guidelines or their significance, and there is often inadequate communication from healthcare providers about the benefits and specifics of the updated ANC model. Past negative experiences with healthcare providers, along with mistrust and inconsistent service quality, may also discourage adherence [9, 10]. Although the ANC8+ recommendation was introduced in 2016, Ethiopia has adopted this model and developed its ANC guidelines in accordance with the WHO 2016 recommendations [11]. Ethiopia formally transitioned from the traditional four‐visit ANC framework to the World Health Organization′s 2016 ANC model, which recommends eight contacts, in early 2022 following the integration of the updated guideline into national maternal health policies [11]. Prior to the 2016 revision of the World Health Organization′s ANC recommendations, Ethiopia implemented the four‐visit ANC model as the standard approach to maternal care [12].

The subsequent change to eight recommended contacts was based on evidence showing that more frequent interactions improve maternal and perinatal outcomes by facilitating earlier identification and management of pregnancy‐related risks [13].

The four‐visit model improved ANC coverage and facilitated gradual gains in maternal health in Ethiopia. However, persistent challenges regarding the timely initiation and completion of care highlighted the necessity for adopting the expanded 2016 ANC model [13]. This study is aimed at assessing nonadherence to the WHO 2016 ANC model and the associated factors among women who are delivering and postpartum in southern Ethiopia.

## 2. Research Questions

2.1. What percentage of delivering and postnatal women in southern Ethiopia failed to complete the recommended eight contacts of the 2016 ANC model?
2.2. Which factors are associated with nonadherence to the WHO 2016 ANC model among delivering and postnatal women in southern Ethiopia?

## 3. Method and Materials

### 3.1. Study Setting and Period

An institution‐based cross‐sectional study was employed at Dilla University Teaching Hospital from August 25, 2024, to October 20, 2024.

### 3.2. Source Population

The source population comprised all laboring and postnatal women who were admitted to the labor and postnatal wards at Dilla University Teaching Hospital.

### 3.3. Study Population

The study population comprised all laboring and postnatal women who were admitted to the labor and postnatal wards at Dilla University Teaching Hospital during the data collection period.

### 3.4. Inclusion Criteria

Pregnant women who were admitted to the labor and postnatal ward during the data collection period were included.

### 3.5. Exclusion Criteria

Pregnant and postnatal women who are severely ill and not willing to respond were excluded from the study.

### 3.6. Sample Size Determination and Sampling Technique

The required sample size is determined by using the single population proportion formula by considering the prevalence of nonadherence to the WHO 2016 ANC model, which is taken from a cross‐sectional study conducted in the South Gondar Zone with a prevalence of 74.3%, a significance level of 95%, and a 5% margin of error [10]. Accordingly, after adding for a 10% nonrespondent rate, a total of 324 was the final sample size. A systematic random sampling technique was then employed. The first laboring and postnatal mother included in the study was selected at random, and subsequent participants were chosen at every second interval.

### 3.7. Dependent and Independent Variables

Nonadherence to the WHO 2016 ANC model served as the dependent variable in this study. The study also considered various sociodemographic variables, including age, residence, religion, educational status, occupation, marital status, the occupation of the husband, and the distance to the health facility. The study analyzed various sociocultural and economic factors, including who makes healthcare decisions, the household income source, family size, access to reproductive and health services, distance from health facilities, and the timing of contact during pregnancy. Additionally, it looked at reproductive and health service factors such as gravidity, parity, and the timing of contact during pregnancy as independent variables.

### 3.8. Data Collection Tool and Procedure

The study examined various sociocultural and economic factors, such as who makes healthcare decisions, the sources of household income, family size, access to reproductive and health services, the distance to health facilities, and the timing of contact during pregnancy. Furthermore, it considered reproductive and health service factors, including gravidity, parity, and the timing of contact during pregnancy, as independent variables.

### 3.9. Data Quality Control

The questionnaire was pretested on 5% of the sample size at Yirgachefe Primary Hospital. Consensus and a common understanding were established among the data collectors regarding the data collection process. Afterward, the data were reviewed for completeness. To ensure the validity and reliability of the data collection instrument, the questionnaire was revised based on the results of the pretest. Data collection was carried out by three diploma‐level midwives in the postnatal and labor wards before patient discharge. This process was supervised by two degree‐level midwives. Data were collected through face‐to‐face interviews and chart reviews, utilizing a structured questionnaire administered by the interviewer.

### 3.10. Operational Definition

Nonadherence to ANC: when a pregnant woman fails to attend or follow through with the recommended ANC visits (having ANC follow‐up less than eight contacts) as advised by healthcare professionals.

Adherence: mothers who complete eight or more ANC contacts recommended by the WHO 2016 ANC model.

Contact: the context of healthcare typically refers to any interaction between a patient and a healthcare provider, such as a doctor, nurse, or other medical professional. This can include in‐person visits, phone calls, emails, or other forms of communication.

Severely ill: pregnant women having life‐threatening health conditions that make them unable to participate in the study.

Decision‐maker for healthcare: the person primarily responsible for determining if household members pursue healthcare, what kind of services they use, and the timing of seeking care.

Head of household: the person recognized by household members as the household head, who is usually responsible for making major decisions and overseeing overall household management.

Principal income generator: the individual who contributes the greatest portion of the household′s income, whether through employment, business activities, or other sources.

### 3.11. Data Processing and Analysis

Data were collected using the Kobo tool and then exported into SPSS Version 25 for analysis. Various data cleaning methods within the software were applied to ensure accuracy and consistency. Descriptive statistical analysis, including simple frequencies, measures of central tendency, and measures of variability, was used to describe the characteristics of the data. Bivariate logistic regression was performed to examine the association between each independent variable and the outcome variable, with a *p* value < 0.25 considered significant. For the multivariable analysis, statistical significance was declared at a *p* value < 0.05 with a 95% confidence interval.

## 4. Results

### 4.1. Sociodemographic Characteristics of Study Participants

Out of 324 eligible women, 308 participated in the study, yielding a response rate of 95.06%. The mean age of study participants was 26.16 ± 5.51 years. The majority, 270 (87.6%), of the women were married; 107 (34.7%) were in the age range of 25–29 years; 94 (30.5%) of the women attended secondary school; 153 (49.7%) participants were housewives; and 160 (51.9%) resided in urban settings (Table 1).

**Table 1:** Sociodemographic characteristics of laboring and postnatal women in Dilla Teaching Hospital, Ethiopia, 2024 (*n* = 308).

Characteristics		Frequency (*n* = 308)	Percentage (%)
Age	< 20	53	17.2
	20–24	54	17.5
	25–29	107	34.7
	30–34	62	20.1
	≥ 35	32	10.4
Religion	Orthodox	63	20.5
	Muslim	39	12.7
	Protestant	206	66.8
Maternal education	No formal education	73	23.7
	Primary	76	24.7
	Secondary	94	30.5
	College and above	65	21.1
Maternal occupation	Housewife	153	49.7
	Farming	18	5.8
	Merchant	33	10.7
	Civil servant	62	20.1
	Other	42	13.6
Marital status	Married	270	87.6
	Single	13	4.2
	Divorced	15	4.8
	Widowed	10	3.2
Husband′s educational status	No formal education	57	18.5
	Primary school	63	20.5
	Secondary school	83	26.9
	College and above	105	34.0
Husband′s occupation	Farmer	56	18.1
	Merchant	68	22.0
	Civil servant	101	32.8
	Other	83	26.9
Residence	Urban	160	51.9
	Rural	148	48.1
Head of household	Wife	35	11.3
	Husband	273	88.6
Decision‐maker	Wife alone	54	17.5
	Wife and husband	207	67.2
	Husband alone	47	15.3
Principal income generator	Wife	25	8.1
	Husband	119	38.6
	Both	147	47.7
	Others	17	5.5
Means of transportation	On foot	132	42.8
	By car	176	57.1
Distance to health facility (km)	< 5 km	242	78.5
	5–10 km	66	21.4
Hours taken to reach the health service	Within 1 h	284	92.2
	1–2 h	13	4.2
	> 2 h	11	3.5
Family size	< 3	158	51.2
	3–4	31	10.0
	≥ 5	119	38.6

### 4.2. Reproductive and Health Service–Related Factors

In this study, 62.3% of study participants were nullipara, 64.6% were multigravida, and 194 (63%) of them started ANC follow‐up before 12 weeks of gestation. In this study, 4.2%, 6.5%, 6.8%, 9.7%, 2.6%, 8.4%, 2.3%, 4.9%, and 54.5% of study participants have no 0, 1, 2, 3, 4, 5, 6, 7, and 8 ANC follow‐ups during this pregnancy, respectively. The most common reason for fewer than eight ANC follow‐ups was discontinuation of service by study participants due to being busy and time‐consuming to get the service (Table 2).

**Table 2:** Reproductive and health service–related factors among laboring and postnatal women in Dilla Teaching Hospital, southern Ethiopia, 2024 (*n* = 308).

Variables		Frequency (*n* = 308)	Percentage (%)	
Parity	Nullipara	192	62.3	
	Primipara	16	5.2	
	Multipara	100	32.5	
Gravidity	Primigravida	109	35.4	
	Multigravida	199	64.6	
Pregnancy status	Planned	260	84.4	
	Unplanned	48	15.6	
Under five children	Yes	201	65.3	
	No	107	34.7	
GA when started ANC	< 12 weeks	194	63	
	12–27 weeks	75	24.3	
	≥ 28 weeks	39	12.7	
Number of ANC contact during this pregnancy	Not	13	4.2	
	One time	20	6.5	
	Two times	21	6.8	
	Three times	30	9.7	
	Four times	8	2.6	
	Five times	26	8.4	
	Six times	7	2.3	
	Seven times	15	4.9	
	Eight and above times	168	54.5	
Reasons for if less than eight ANC contact	Discontinue of service	73	52.1	*n* = 140
	Delay registration	67	47.9	
Reasons for discontinuation of ANC contact	They do not tell me when to come back	9	12.3	*n* = 73
	Due to distance	17	23.2	
	Being busy	29	39.7	
	The service was not attracted	6	8.2	
	Time consuming to get the service	12	16.4	

### 4.3. Nonadherence of WHO 2016 ANC Models

**Figure 1:**
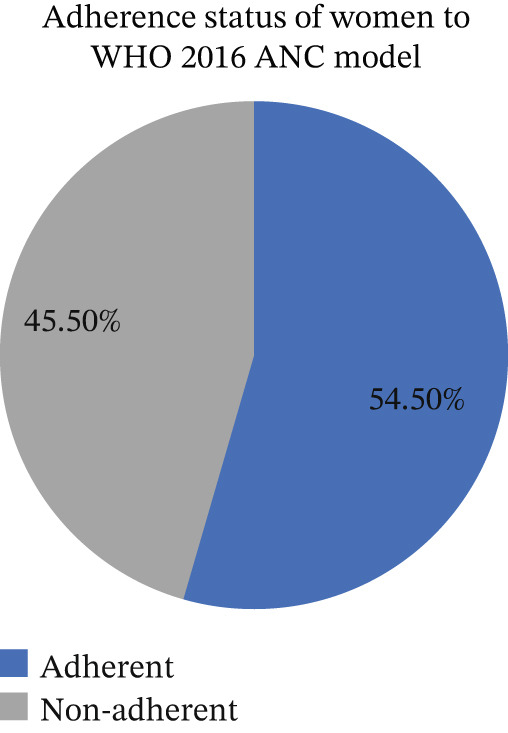
Nonadherence to WHO 2016 antenatal care models during pregnancy among laboring and postnatal women in Dilla Teaching Hospital, Ethiopia, 2024.

In this study, about 45.5% (95% CI: 42.12, 56.3) of respondents were nonadherent to the WHO 2016 ANC model (Figure 1). The most common reasons for nonadherence to the WHO 2016 ANC model include discontinuation of service by the pregnant women (73, or 23.7%) and delayed registration (67, or 21.7%), respectively (Table 2).

### 4.4. Factors Associated With Nonadherence of 2016 WHO ANC Model

In the bivariable logistic regression analysis, lack of formal education, households headed by the husband, the husband being the primary income generator, and traveling to the health facility on foot showed significant associations with nonadherence to the 2016 WHO ANC model. In the final multivariable logistic regression model, women with no formal education, women who reside in rural settings, women whose husbands were head of the household, and women whose husbands were the household′s primary income generator were significantly associated with nonadherence to the WHO 2016 ANC model (*p* < 0.05) (Table 3).

**Table 3:** Bivariable and multivariable logistic regression of nonadherence to the WHO 2016 antenatal care model and associated factors among delivering and postnatal women in southern Ethiopia, 2024.

Variables		ANC		COR (*C* *I* = 95*%*)	AOR (*C* *I* = 95*%*)
		Nonadherence	Adherence		
Residence	Rural	87 (63.5%)	50 (36.5%)	4.2 (2.8–5.93)	2.4 (1.14–3.42) ^∗^
	Urban	50 (29.2%)	121 (70.8%)	Reference	Reference
Maternal education	No formal education	53 (72.6%)	20 (27.4%)	10.80 (8.38–13.77)	2.174 (1.03–3.55) ^∗^
	Primary	42 (55.3%)	34 (44.7%)	5.03 (3.86–8.331)	0.71 (0.08–1.510)
	Secondary	29 (30.9%)	65 (69.1%)	1.811 (0.192–3.678)	0.95 (0.213–1.950)
	College and above	13 (20.0%)	53 (80.0%)	Reference	Reference
Head of household	Wife	12 (57.1%)	9 (42.9%)	Reference	Reference
	Husband	116 (42.0%)	160 (58.0%)	1.83 (2.67–3.45)	11.53 (1.024–38.6) ^∗^
	Both	9 (81.8%)	2 (18.2%)	3.27 (0.18‐6.63)	1.95 (0.001‐4.9)
Income generator	Wife	11 (57.9%)	8 (42.1%)	Reference	Reference
	Husband	58 (52.3%)	53 (47.7%)	1.25 (1.044–4.50)	2.01 (1.009–4.08) ^∗^
	Both	60 (35.5%)	109 (64.5%)	2.49 (0.529–4.38)	0.9 (0.006–1.73)
	Other	8 (88.9%)	1 (11.1%)	0.68 (0.02–2.29)	7.21 (0.035–13.423)
Means of transportation	By car	70 (42.2%)	96 (57.8%)	Reference	Reference
	On foot	70 (55.6%)	56 (44.4%)	0.58 (0.01–2.73)	1.01 (0.32–2.597)
Planned pregnancy	Yes	115 (41.4%)	163 (58.6%)	Reference	Reference
	No	22 (73.3%)	8 (26.7%)	0.25 (0.115–1.61)	0.97 (0.056–1.6)

Abbreviations: AOR, adjusted odds ratio; COR, crude odd ratio.

^∗^Significant at *p* value < 0.005.

## 5. Discussion

The WHO issued new guidelines in 2016 regarding the number of ANC contacts required for a positive pregnancy experience, increasing the minimum of four visits to eight ANC contacts, with the first contact occurring in the first trimester and increased contacts occurring during the third trimester [14]. However, increasing the number of ANC contacts might not be enough unless nonadherence status and factors are determined. The main aim of this study was to assess the magnitude of nonadherence to the new WHO 2016 ANC model among postnatal and laboring women.

In this study, we found that 45.5% of pregnant women were nonadherent to the recommended WHO 2016 ANC model. This finding is in line with other studies done in Cameroon 56% [15]. However, the present study showed a lower rate of nonadherence to the WHO 2016 ANC model compared with studies conducted in Sierra Leone 73.9% [16], Bangladesh 94% [17], Myanmar 82% [18], Zambia 98.7% [19], Gambia 95.7% [20], Guinea 96.5% [21], Mali 96.5% [22], Cameroon 91.1% [23], and Nigeria 82.6% [24]. The possible reason for lower magnitude nonadherence in this study might be due to the presence of exempted maternal health services and the difference in study periods.

Conversely, our study has a higher rate of nonadherence to the 2016 WHO ANC model than a multicountry study in LMICs [25], DHS data from Nigeria 20.3% [26], and Benin 8% [27]. The justification for these variations might be the difference in study population and different time for initiation of the 2016 WHO ANC model. Failing to adhere to recommended ANC can significantly compromise the health of both mothers and their newborns. Missing scheduled ANC visits reduces opportunities for the early identification and treatment of pregnancy‐related complications such as preeclampsia, gestational diabetes, anemia, and infections, which, if left unaddressed, can become life threatening. It also limits access to critical preventive services, including immunizations, nutritional guidance, supplementation, and education on birth preparedness and danger signs. For infants, inadequate ANC increases the likelihood of preterm delivery, low birth weight, neonatal infections, and higher newborn mortality.

This study showed that women who reside in rural settings were 2.4 times more nonadherent to the WHO 2016 ANC model than those who reside in urban settings (95% CI: 3.14–5.42). Similarly, studies conducted in South Gondar [10], and Bangladesh [17] showed that women residing in rural settings were more likely to be nonadherent than their counterparts. This may be due to rural areas often facing a shortage of health facilities, longer travel distances to clinics, inadequate transportation, and a limited number of skilled healthcare providers, all of which make attending multiple ANC visits challenging. Women in these settings also tend to have lower levels of education and less access to health information through media or community outreach, which reduces their awareness of the importance of early and consistent ANC visits. Financial constraints, including lower household income and the indirect costs of transportation and time away from farming or household duties, further hinder adherence. Additionally, cultural practices and reliance on traditional birth attendants are more common in rural communities, often delaying engagement with formal healthcare services. Altogether, these structural, economic, and social factors create significant barriers to completing the recommended eight ANC contacts.

In this study, women with no formal education were 2.17 times more likely to be nonadherent to the WHO 2016 ANC model compared with women who had a college education or higher (95% CI: 1.03–3.55). Education plays a crucial role in shaping health literacy; women who lack formal schooling often have limited awareness of the importance of early initiation of ANC and regular attendance, are less familiar with pregnancy danger signs, and may struggle to understand health information provided by healthcare professionals. As a result, they are more likely to initiate care late and attend fewer visits than recommended. In addition, low educational attainment is frequently associated with poverty and limited access to resources, creating financial and practical barriers such as transportation costs and competing time demands.

This study revealed that women whose husbands were the primary income generators had twice the odds of nonadherence to the WHO ANC model (95% CI: 1.009–4.08). A possible explanation is that women may lack financial autonomy; when husbands control household finances, wives must request money to cover transportation to health facilities and registration fees. Similarly, women whose husbands were the heads of their households were 11.53 times more likely to be nonadherent to the WHO ANC model compared with their counterparts (95% CI: 1.024–38.6). Women living in households where husbands are the primary decision‐makers are more likely to be nonadherent to the World Health Organization ANC model, as male‐dominated authority can restrict women′s ability to seek timely and repeated care. In such contexts, women often require their husband′s approval to access health services, and if the husband lacks awareness of the importance of regular ANC visits or places greater emphasis on financial or work obligations, attendance may be delayed or reduced. Moreover, reliance on husbands for financial resources can limit access to transportation and other indirect costs associated with multiple visits. Cultural norms that position men as the main decision‐makers may also hinder open discussion of maternal health needs, further affecting continuity of care. Consequently, limited autonomy, economic dependence, and insufficient partner support together contribute to poor adherence to the recommended ANC schedule.

## 6. Conclusion and Recommendations

In this study, we found that 140 (45.5%) postnatal and laboring women were nonadherent to the newly recommended WHO 2016 ANC contacts. Maternal education, having a husband as the primary income generator, the husband being the head of the household, and residing in rural settings were all significantly associated with nonadherence to the WHO 2016 ANC model. Therefore, to reduce nonadherence, efforts should focus on women′s economic empowerment and education, with particular emphasis on raising awareness about the importance of frequent ANC visits. Additionally, the Ethiopian government should work to improve access to health facilities to help achieve the Sustainable Development Goals (SDGs).

## 7. Limitation of the Study

The current study employed a quantitative design and did not explore how nonadherence to ANC affects the continuum of care, nor did it assess adverse clinical outcomes such as maternal and neonatal outcomes. These are the limitations of our study.

**Nomenclature**
ANC: antenatal care
CI: confidence interval
EDHS: Ethiopian Demographic and Health Survey
LMIC: low‐ and middle‐income countries
MMR: maternal mortality ratio
OR: odds ratio
SDG: Sustainable Development Goal
SPSS: Statistical Package for the Social Sciences
SSA: sub‐Saharan Africa
WHO: World Health Organization

## Author Contributions

Zerihun Figa conceived the study and wrote the original draft of the manuscript. Kassa Genetu and Anteneh Gashaw analyzed the data and interpreted them. Genet Mekuye and Addisu Getnet reviewed the draft manuscript for intellectual content and participated in the revision.

## Funding

No funding was received for this manuscript.

## Disclosure

All the authors read and approved the final version of the manuscript.

## Ethics Statement

The Institutional Review Board (IRB) of Dilla University provided ethical approval before it was given to the study hospital and the relevant organizations for formal approval. Each pregnant woman was provided written informed consent right before the actual data collection, following a discussion and explanation of the study′s goals and overall approach. For participants who were unable to read or write, the consent form was read aloud in the presence of a witness (a friend, relative, or an independent individual not affiliated with the research team), and the witness signed to confirm consent. For participants whose primary language was not Amharic, the consent form was translated into the local language and back‐translated to ensure accuracy and consistency. Participants were informed of their right to withdraw from the study at any time without consequences. Confidentiality and privacy were strictly maintained, and data were not disclosed to any third party other than the investigators. Finally, data collection was conducted in a manner that respected the rights, culture, norms, and ethical considerations of the participants. All procedures were carried out in accordance with institutional guidelines and the Declaration of Helsinki.

## Consent

The authors have nothing to report.

## Conflicts of Interest

The authors declare no conflicts of interest.

## Data Availability

The data underlying the results of the study are available and can be accessed from the corresponding author, and can be shared upon reasonable and legal request via kassagene2@gmail.com.
